# A Rare Differential Diagnosis of a Nasal Tumor: Case Report and Literature Review

**DOI:** 10.1155/2015/318620

**Published:** 2015-10-19

**Authors:** S. Burkart, U. Schoenenberger

**Affiliations:** Department of Otorhinolaryngology, Cantonal Hospital St. Gallen, 9007 St. Gallen, Switzerland

## Abstract

Vascular leiomyomas or angioleiomyomas are rare tumors that can be found in the nasal cavity. The etiology of angioleiomyoma remains poorly understood and there are several hypotheses to explain the origin of sinonasal leiomyoma. We here describe the clinical and histological findings in a case study along with the feasibility of surgical treatment using a radiofrequency instrument. In particular, we describe the case of an adult patient with recurrent epistaxis because of a nasal angioleiomyoma and the performed treatment in the form of complete surgical excision. Radiological imaging is a helpful tool to give an indication of the extension of the tumor, as well as for the proper planning of the surgical approach. Either MRI or CT scans are found to be best suited for this purpose. This case report recommends the complete surgical excision of the angioleiomyoma, by either an endoscopic or an open procedure. This can be safely performed using a radiofrequency instrument as shown in this case with no recurrence during a follow-up of 12 months.

## 1. Introduction

Vascular leiomyoma (VL) or angioleiomyoma comparable to the one described in this study is extremely rare. Only 8.5% to 10% of VL are found in the head and neck area and, among these, only 3% occur in the nasal cavity [1, 2]. Intranasal leiomyomas were first reported by Maesaka et al. in 1966 [3]. We describe the clinical and histological findings and the feasibility of surgical treatment using a radiofrequency instrument.

## 2. Case Report

A 45-year-old man reported to his general practitioner with recurrent and strong, but self-limiting, left-sided epistaxis over the preceding 2 to 3 months, each time lasting up to 10 minutes. He also felt an intermittent, dull pain on the left side of his nose and the strange sensation of a slowly growing mass inside his nose, but neither nasal obstruction nor hypesthesia of the face. An excision under local anesthesia was then attempted. The effort was abandoned because of strong bleeding, a nosepad was applied and then the patient was referred as an emergency to our clinic. Upon arrival the bleeding had stopped.

The personal history showed an appropriately treated arterial hypertension and no regular alcohol or nicotine consumption or substance abuse. There was no high incidence of cancer in the family history.

A clinical examination showed a healthy albeit obese man with a marginally elevated blood pressure level. After removal of the nosepad and suction cleaning, the nasal inspection showed a well-defined tumor of the lateral nasal wall on the anterior face of the inferior turbinate, approximately 10 mm in size. The tumor surface exhibited several incisions. Anterior and posterior rhinoscopy revealed a slight deviation of the nasal septum, but no other intranasal pathology. The external nose showed a slight, though distinctly palpable swelling in the area between the lateral cartilage and the nasal bone on the left side.

**Figure 1:**
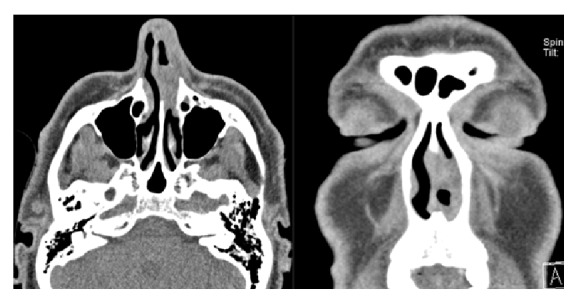
Contrast enhanced CT scan showing a lesion of the inferior turbinate on the left side. Minimal contrast enhancement on the tumor margins.

**Figure 2:**
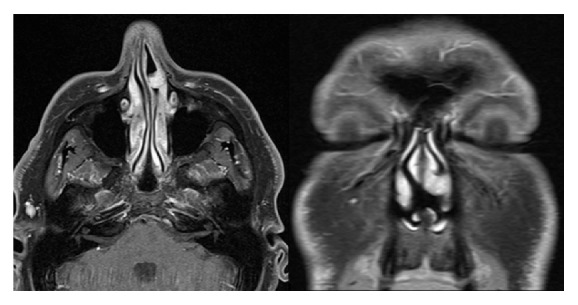
Contrast enhanced MRI, T1-weighted showing a single nodular lesion emanating from the lateral nasal wall and extending to the anterior face of the inferior nasal turbinate on the level of the piriform aperture without any sign of infiltrative growth.

To further investigate the repeatedly bleeding endonasal mass a CT scan with contrast was conducted. It showed a tumor of the inferior turbinate, only marginally contrast enhancing, 24 mm in the largest diameter. No bony erosion was reported, and an infiltration of the lateral nasal cartilage could not be ruled out (Figure 1). A biopsy under local anesthesia revealed the diagnosis of angioleiomyoma. To differentiate the tumor growth and the involvement of nasal cartilage, a MRI scan was ordered (Figure 2). The scan showed a 9 × 11 × 8 mm (*W* × *H* × *D*) sized, nodular, contrast enhancing lesion emanating from the lateral nasal wall and extending to the anterior face of the inferior nasal turbinate on the level of the piriform aperture without any sign of infiltrative growth.

**Figure 3:**
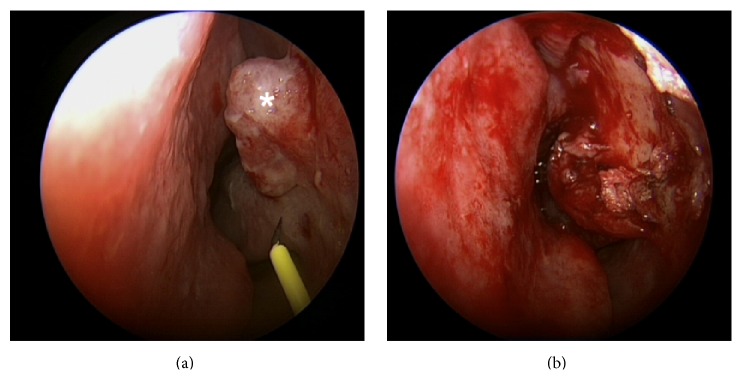
Intraoperative image of the angioleiomyoma (*∗*) before (a) and after (b) complete resection.

**Figure 4:**
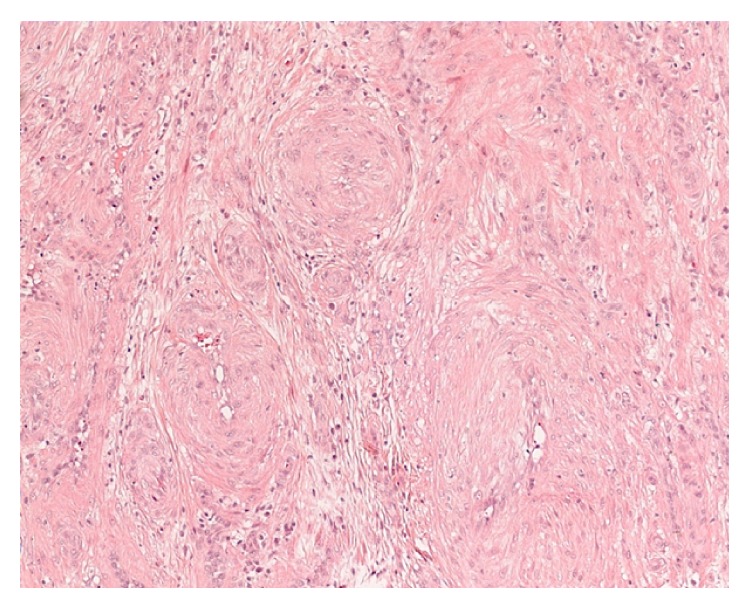
Histopathological examination: proliferation of smooth muscle cells permeated by thick-walled vessels with narrow lumens. No evidence of pleomorphism, mitosis, nuclear atypia, or necrosis. Hematoxylin and eosin stain; original magnification ×100.

A transnasal endoscopic tumor resection was performed under general anesthesia using a radiofrequency instrument (Ellman Surgitron) (Figure 3). Intraoperative blood loss was minimal and no complications occurred. Nasal packing was applied and removed on the first postsurgical day. Postoperative nasal endoscopy showed a well-healing wound with no sign of infection. The histological examination confirmed the diagnosis of angioleiomyoma (Figure 4). During the clinical follow-ups conducted at six and twelve months after the surgery, a nasal endoscopy showed no sign of tumor recurrence and the patient remained symptom-free.

## 3. Discussion

Vascular leiomyomas are benign neoplasms that are rarely found in the head and neck region. The presenting symptoms are nonspecific and usually characterized by nasal obstruction, epistaxis, and nasal discharge. Local pain is not common in sinonasal leiomyoma but has also been described. Vascular leiomyomas occur as small, painless masses in the superficial layer of the skin or mucosa. Auricle, nose, lip, and neck are the most common areas of occurrence [4].

These tumors can affect persons of any age, but they are more common in people between 30 to 60 years of age with a female predominance (female to male ratio of 2 to 1) [5].

While the etiology of sinonasal leiomyoma remains uncertain, there are three dominant hypotheses to explain the origin of sinonasal leiomyoma.

They could stem from aberrant undifferentiated mesenchymal or smooth muscle elements in the blood vessel wall [6]. In the nasal vestibule, they might arise from erector pilae or sweat gland muscles [7]. The observation of many tumors located in nasal turbinates seems to support this hypothesis; these areas are relatively abundant with blood vessels and smooth muscles.

Some authors suggest that vascular leiomyoma could be a vascular malformation or a progressive development of smooth muscle proliferation from hemangioma, to angioma with much muscle, to leiomyoma with many vessels, and to solid leiomyoma [2]. The inferior nasal turbinate, the nasal septum, and the nasal vestibule are reported as the most common sites of occurrence [5].

Marioni et al. [8] hypothesized that the tumor growth might be hormone-dependent due to the reported female predominance and the increased pain during pregnancy or the menstrual cycle in patients described elsewhere. Sex hormone dependence is still controversial regarding nasal vascular leiomyoma. More studies are needed to clarify the influence and mechanism of action of sex hormones on this tumor type.

The World Health Organization (WHO) classified leiomyoma into three groups: nonvascular leiomyoma, vascular leiomyoma, and epithelioid leiomyoma. Morimoto divided angiomyoma into three histologic subtypes in 1973: solid or capillary, cavernous, and venous. The vascular subtype is the most common one and angiomyoma of the head and neck usually occurs as a venous or cavernous type [2]. The solid type has smooth muscle bundles that intertwine and surround the vascular slit-like channels [9]. The cavernous type has dilated vascular channels with smaller amounts of smooth muscle [9]. The venous type has vascular channels with thick muscular walls that are easily discerned from smooth muscle bundles [9].

The histopathological differential diagnosis of angioleiomyoma includes hemangioma, angiofibroma, myopericytoma, fibromyoma, and leiomyosarcoma [8]. Sinonasal tumors can be of epithelial or mesenchymal origin. Epithelial tumors are the most common and originate from the epithelial lining, accessory salivary glands, neuroendocrine tissue, and olfactory epithelium. Mesenchymal tumors derive from the supporting tissue [10].

Inverted papillomas and osteomas are the most common benign tumors followed by fibrous dysplasia and neurogenic tumors, for example, schwannomas.

Squamous cell carcinomas are the most common malignant sinonasal tumors (80%), followed by adenocarcinoma. Chondrosarcoma, lymphoma, salivary gland tumors, neuroendocrine tumors, and mucosal malignant melanoma represent a further differential diagnosis.

Radiological imaging, for example, MRI and/or CT scans, represents an helpful tool for visualizing the extension of the tumor and for the proper planning of the surgical approach, but there are no particular imaging techniques that specifically characterize the VL [11]. Nevertheless, images may be helpful in differentiating deeply seated vascular leiomyoma from malignant or other benign tumors, such as lipoma or fibroma [12, 13]. Surgery is the treatment of choice for this benign tumor. The surgical approach depends on the size, the location, the extension of the tumor, and the experience of the surgeon. Different surgical techniques are well known from the surgery of the turbinates (e.g., resectioning, laser surgical procedures, and coagulation procedures). In most patients, transnasal endoscopic excision as shown in our case can be performed successfully because many of the tumors are limited to the sinonasal cavity. The presented technique of excision with a radiofrequency instrument has many advantages, for example, clear surgical field and less hemorrhage, and can be safely performed by an experienced surgeon in a two-hand technique.

There are no reports of recurrence after total excision [14, 15]. Yang et al. [11] mention one patient with recurrent leiomyoma two years after surgery. This was probably due to an incomplete excision of the tumor margin and the adjacent bony shell. Thus, the chance of local recurrence is mainly related to incomplete excision at the time of the initial surgery. Malignant variants of this neoplasm have been reported; however, they are rare. It appears that the absence of mitosis is the most useful histological indicator of a benign lesion [16]. Surgical excision accompanied by a histological study is the only way to confirm the diagnosis [9]. Most vascular leiomyomas can be correctly diagnosed under the microscope with conventional H&E staining. Special stains for smooth muscle cells are sometimes necessary to differentiate VL from other spindle cell tumors such as hemangioma, angiofibroma, fibroma, and angiomyolipoma [17].

## 4. Conclusion

Angioleiomyoma in the nasal cavity is extremely rare but an important differential diagnosis in a patient with a nasal tumor. Complete surgical excision is recommended either by an endoscopic or by an open procedure. Attention should be paid to intra- and postoperative hemorrhage due to the hypervascularity of this neoplasm. In our case of a VL originating from the anterior border of the inferior turbinate, we demonstrated that the endoscopic approach with a radiofrequency instrument resulted in a complete and safe removal of the mass with no recurrence during a period of 12 months.
